# REVEALS—a longitudinal cohort study of multifaceted respiratory assessment in ALS

**DOI:** 10.1080/21678421.2024.2359556

**Published:** 2024-06-06

**Authors:** James Rooney, Deirdre Murray, Dara Meldrum, Ammar Al-Chalabi, Tommy Bunte, Theresa Chiwera, Mutahhara Choudhury, Adriano Chio, Lauren Fenton, Jennifer Fortune, Lindsay Maidment, Umberto Manera, Christopher J. McDermott, Myrte Meyjes, Rachel Tattersall, Maria Claudia Torrieri, Philip Van Damme, Elien Vanderlinden, Claire Wood, Leonard H. van den Berg, Orla Hardiman

**Affiliations:** 1 Academic Unit of Neurology, Trinity College Dublin, Dublin, Ireland; 2 Institute and Clinic for Occupational, Social and Environmental Medicine, University Hospital, LMU Munich, Munich, Germany; 3 Beaumont Hospital, Dublin, Ireland; 4 Department of Basic and Clinical Neuroscience, King’s College London, Maurice Wohl Clinical Neuroscience Institute, London, UK; 5 Department of Neurology, King’s College Hospital, London, UK; 6 UMC Utrecht, Utrecht, The Netherlands; 7 ALS Center, ‘Rita Levi Montalcini’ Department of Neuroscience, University of Turin, Turin, Italy; 8 Neurology 1, Azienda Ospedale Università Città della Salute e della Scienza, Turin, Italy; 9 Sheffield Teaching Hospitals NHS Foundation Trust, Sheffield, UK; 10 Department of Neuroscience, Sheffield Institute for Translational Neuroscience, University of Sheffield, Sheffield, UK; 11 Neurology Department, KU Leuven, University Hospitals Leuven, Leuven, Belgium, and; 12 Department of Neuroscience, KU Leuven, Leuven Brain Institute and VIB Center for Brain & Disease Research, Leuven, Belgium

**Keywords:** Respiratory measurement, ALS disease onset, longitudinal decline

## Abstract

**Objective:**

To systematically assess decline in respiratory measures in amyotrophic lateral sclerosis (ALS) and to examine the impact of sex, disease onset type and baseline morbidity on progression.

**Methods:**

The REVEALS study (Registry of Endpoints and Validated Experiences in ALS) was conducted between April 2018 and February 2021 in six European ALS centers. Slow and forced vital capacity (S/FVC), sniff nasal inspiratory pressure (SNIP), peak cough flow, amyotrophic lateral sclerosis functional rating scale-revised (ALSFRS-R), and respiratory morbidity were collected. Data were analyzed using a Bayesian multiple outcomes random effects model.

**Results:**

Two hundred and eighty participants had a median of three assessments (IQR 2.0, 5.0) over a median of 8 months (IQR 2.3, 14.1). There were 974 data collection timepoints. Differences in respiratory measures and rates of decline between disease-onset and sex subgroups were identified. Females had lower scores in all respiratory measures and females with bulbar onset ALS had faster decline compared with other sub-groups. These differences were not detected by the ALSFRS-r respiratory subscale. Dyspnea, orthopnea, and a higher King’s stage at baseline were associated with lower respiratory scores throughout follow-up, while having a regular productive cough at baseline was associated with lower peak cough flow scores.

**Conclusion:**

Respiratory function declines more quickly in females with ALS compared with males when measured by FVC, SVC, SNIP, or PCF, but not the ALSFRS-R respiratory sub-score. Higher baseline King’s staging and the presence of clinical respiratory symptoms at baseline were associated with worse respiratory function. The ALSFRS-R respiratory sub-score is poorly correlated with objective respiratory measurements.

## Introduction

Amyotrophic lateral sclerosis (ALS)/motor neuron disease (MND) results in progressive decline in respiratory muscle strength and ultimately ventilatory failure (1). Symptoms include shortness of breath and difficultly coughing (2). Declining respiratory function is a prognostic indicator in ALS (3) and clinical guidelines advocate frequent assessment (2,4). Pulmonary function tests including forced vital capacity (FVC), slow vital capacity (SVC), sniff nasal inspiratory pressure (SNIP) and peak cough flow assess decline and guide intervention with non-invasive ventilation (NIV) and cough augmentation devices (2,5,6). In clinical trials, the amyotrophic lateral sclerosis functional rating scale-revised (ALSFRS-R), which includes a respiratory subscale, is used as a primary endpoint (7), but the ability of the respiratory sub-scale to adequately assess respiratory function has been questioned (8), and it does not consider ability to clear bronchial secretions.

Disease heterogeneity presents a challenge for clinical care and trials of new treatments (9) as the disease course differs between patients with spinal- and bulbar-onset disease (10), with overall incidence higher in males, and more females presenting with bulbar-onset disease (11,12). Specific genetic phenotypes manifest in different respiratory decline profiles in sex-based subgroups (13,14), but patterns and rates of decline in respiratory measures in disease-onset and sex subgroups require further definition. Differences in presentation of respiratory dysfunction in males and females have not been defined, and insights into patterns of decline across subgroups is important both for clinical prognostication and for stratification of participants in clinical trials.

Forced and slow vital capacity, commonly used in clinical trials, measure the maximum volume of air that can be exhaled from the lungs during a complete expiration from a position of full inspiration. Vital capacity is a global measure of respiratory function, involving multiple muscle groups and passive recoil of a distended chest. These measures are valid and reliable and have stringently applied international standards (15), as well as guidelines specific to ALS (16). Additional tests including SNIP and peak cough flow are used in clinical practice to guide intervention (2,4,5) and have been used in clinical trials (17). SNIP, measures inspiratory muscle strength and is reported in centimeters of water (cmH_2_O), peak cough flow measures the maximal flow of air achieved during a cough and is an indication of the ability to effectively clear secretions (18).

The REVEALS study (Registry of Endpoints and Validated Experiences in ALS) was conducted between April 2018 and February 2021 in six European specialist ALS centers (Beaumont Hospital, Dublin, Ireland; King’s College Hospital, London, UK; University Medical Center, Utrecht, The Netherlands; Azienda Ospedale Università Città della Salute e della Scienza, Turin, Italy; Sheffield Teaching Hospitals, Sheffield and University Hospitals Leuven, Belgium). The aim of the study was to systematically assess longitudinal change in key respiratory measures in ALS and to examine the relationships between these measures and respiratory morbidity.

Using multivariate modeling, we have previously shown that each of the measures, FVC, SVC, SNIP, and peak cough flow demonstrated decline over time, with differential decline in bulbar and spinal-onset patients more clearly demonstrated in SNIP and peak cough flow than in FVC and SVC. We found that although FVC and SVC were strongly correlated, SNIP was only moderately correlated with FVC and SVC, reflecting the assessment of a different aspect of respiratory function using this test (19).

Here, we report the decline in these respiratory measures, including the ALSFRS-R respiratory sub-scale, assessed concurrently over time. The impact of possible confounders on the patterns of decline in respiratory measurements, in particular the patterns evident in males and females with spinal- and bulbar-onset ALS is examined. We examine the impact of respiratory symptoms and history at baseline on the measures. We also re-examined the relationships between the respiratory measures.

## Methods

### Study design

This was a longitudinal, observational study conducted in a real-world clinic setting at six clinical sites (19). Patients attending a participating clinic, with a confirmed diagnosis of spinal or bulbar-onset ALS were eligible to participate. Additional inclusion criteria were: ALS King’s stage 2 or 3 at recruitment (20), the ability to provide informed consent, the ability to complete respiratory tests (defined as ability to generate consistent scores, of two valid scores within 10% in SVC and FVC), and the ability to correspond remotely, either independently or with the assistance of a carer. Progression of ALS to the point of using NIV at the time of recruitment or another active respiratory condition (COPD, bronchiectasis, lung cancer, etc.) was exclusion criteria. Reassessment at irregular intervals was acceptable in light of the “real world” design of the study, although 3-monthly assessments in line with guidelines on clinical review were targeted. All assessors completed training in outcome measurement procedures and received regular site visits, including data collection observation, which ensured consistency of the protocol across the sites.

### Outcome measures

Four respiratory outcome measurements were collected in the same order at each visit with the maximum score recorded for analysis; SNIP (cmH_2_O) (at least 10 trials), peak cough flow (liters (L)/min) (at least six trials), SVC (L and % predicted), and FVC (L and %predicted) (3–5 trials). The %predicted score of FVC was calculated using global lung function initiative (GLI) reference equations (21) and the %predicted for SVC was calculated using the ratio provided by FVC [%pred SVC = (FVC %pred × SVC (L)) ÷ FVC (L)]. The procedures for these assessments have been described in detail previously (19) and adhered to standard operating procedures (16). Potential issues that may affect successful completion of SNIP (nasal surgery or deviation, congestion or other issues) were recorded.

In addition, demographic and clinical variables were collected at baseline including site of ALS onset (spinal/bulbar), date of onset, date of diagnosis, sex, height, weight, King’s staging, history of chest infections, smoking history (ever vs. never smoked), and the presence of orthopnea, dyspnea at rest, dyspnea when active or having a regular productive cough (defined as regularly needing to clear phlegm). The ALSFRS-R was completed (16) and the ALSFRS-R respiratory sub-scale was calculated. Chest infection incidence and symptoms were collected at clinic visits and prospectively via fortnightly text message.

Data were collected from April 2018 until February 2020 at which time the Covid-19 pandemic affected in-person respiratory measurement. In-person assessment recommenced in Leuven, and in a limited capacity in Utrecht in July 2020, but remained suspended in other sites. Collection of chest infection incidence continued via fortnightly text messages in most sites and some sites collected ALSFRS-R and other subjective questions by phone. The study concluded as planned in February 2021.

### Statistical methods

A descriptive analysis of participants was performed and correlations between outcome measures were calculated for the entire cohort and stratified by site of onset. We refit the previous Bayesian multiple outcomes random effects model (19) to the updated dataset. Random intercepts and random slopes (over time) per individual were included for each outcome (using the Gaussian family for outcome variables), while fixed effects were included for the time from baseline (where the baseline was defined as study enrollment date), site of onset and sex in interaction with time from baseline, and study site. We updated the model to recode site of onset and sex into a single four level variable (“Male spinal-onset”, “Male bulbar-onset”, “Female spinal-onset”, and “Female bulbar-onset”) to allow for differing rates of decline by sex as well as by site of onset. In addition, the model included terms for correlation both between and within individuals and for residual correlation between outcomes. A total of 3500 model iterations were run and model convergence and fit were assessed by assessing the bulk effective sample size and tail effective sample size, and posterior predictive checks. Models were fit using absolute value for FVC and SVC (i.e. measured in liters) and using percent predicted FVC and SVC, with all other parameters the same as for the best fit model selected in the interim analysis (19). Visit timepoints missing any respiratory outcome measure, or individuals missing any explanatory variables were excluded from the models.

The base model was extended to include the ALSFRS-R respiratory sub-score as an additional Gaussian outcome variable. This model was also fit using absolute value for FVC and SVC (i.e. measured in liters) and using percent predicted FVC and SVC.

The model was further extended to include additional clinical variables of particular interest as fixed effects. To avoid reverse causality, investigational explanatory variables included baseline variables: staging, history of respiratory tract infections, smoking history, orthopnea, dyspnea at rest, dyspnea when active or having a regular productive cough. These models were fit using the %predicted FVC and SVC scores.

*Software*: R statistical software 4.3.2 with additional packages was used for data preparation and descriptive analysis (22–26), and R packages brms (27), tidybayes (28), bayesplot (29), and Stan software version 2.29.2 were used to fit and assess Bayesian models. Analysis code is available on Github: https://github.com/jpkrooney/REVEALS_Final_BayesMultivariateAnalysis, and archived on Zenodo: https://zenodo.org/doi/10.5281/zenodo.10863865

## Results

Table 1 summarizes the baseline characteristics of REVEALS participants. Compared with the interim analysis (19), the median follow-up time increased from 6 to 12 months. In total, there were 974 in-clinic data collection timepoints, of which 138 (14.2%) were missing at least one respiratory assessment (SNIP 9.2%, SVC 12.5%, FVC 11.8%, and PCF 9.4% missing). Exclusion of timepoints with missing outcomes resulted in 11(4%) of participants being excluded from models.

**Table 1.** Baseline characteristics of the REVEALS study stratified by recruitment site.

	Total cohort	Dublin	London	Leuven	Sheffield	Turin	Utrecht	*p* Value*
*N*	280	63	22	59	22	56	58	
Female, *n* (%)	93 (33.2)	22 (34.9)	9 (40.9)	21 (35.6)	8 (36.4)	17 (30.4)	16 (27.6)	
Male, *n* (%)	187 (66.8)	41 (65.1)	13 (59.1)	38 (64.4)	14 (63.6)	39 (69.6)	42 (72.4)	0.845
Spinal-onset, *n* (%)	227 (81.1)	53 (84.1)	16 (72.7)	49 (83.1)	21 (95.5)	43 (76.8)	45 (77.6)	
Bulbar-onset, *n* (%)	53 (18.9)	10 (15.9)	6 (27.3)	10 (16.9)	1 (4.5)	13 (23.2)	13 (22.4)	0.316
King’s 2, *n* (%)	167 (59.6)	50 (79.4)	15 (68.2)	24 (40.7)	13 (59.1)	37 (66.1)	28 (48.3)	
King’s 3, *n* (%)	113 (40.4)	13 (20.6)	7 (31.8)	35 (59.3)	9 (40.9)	19 (33.9)	30 (51.7)	
Height in cm (mean (SD))	172.10 (10.05)	170.55 (8.99)	170.56 (8.58)	170.19 (9.71)	172.82 (12.58)	169.71 (8.57)	178.24 (10.07)	<0.001
*N* missing height	3	3	0	0	0	0	0	
Weight in kg (mean (SD))	74.67 (14.05)	76.28 (13.20)	75.00 (15.06)	73.72 (14.51)	76.15 (17.49)	67.01 (11.47)	81.00 (11.77)	<0.001
*N* missing weight	14	11	0	0	3	0	0	
Age at diagnosis (mean (SD))	61.85 (11.85)	63.26 (12.10)	60.43 (13.28)	61.61 (11.99)	56.49 (12.69)	64.37 (10.85)	60.70 (11.05)	0.115
Median diagnostic delay (SD)	9.99 [6.67, 16.33]	9.28 [5.89, 16.13]	ND	9.26 [6.46, 12.80]	ND	11.99 [6.97, 18.25]	10.87 [6.80, 16.33]	0.404
Months from onset to study baseline, median (SD)	19.42 [11.65, 35.12]	17.71 [9.58, 28.48]	ND	26.28 [12.07, 55.72]	ND	21.49 [14.92, 35.43]	17.26 [11.40, 26.60]	0.056
Median in-clinic follow-up time [IQR]	8.0 [2.3, 14.1]	5.8 [2.1, 11.6]	0.00 [0.0, 0.6]	11.9 [5.3, 23.9]	4.1 [0.0, 7.4]	8.9 [2.7, 13.4]	12.3 [3.4, 5.9]	<0.001
Median remote follow-up time [IQR]	12.0 [4.9, 18.1]	15.9 [10.5, 25.0]	4.7 [3.8, 10.5]	15.6 [8.6, 26.6]	9.1 [2.7, 11.9]	9.7 [4.5, 15.7]	13.9 [5.3, 16.5]	<0.001
Median number of assessments per individual [IQR]	3.00 [2.0, 5.0]	3.00 [2.0, 4.0]	1.00 [1.0, 2.0]	4.00 [3.0, 7.0]	2.00 [1.0, 2.75]	4.00 [2.0, 4.0]	4.00 [2.0, 6.0]	<0.001
Mean (SD) FVC in %pred at baseline	79.54 (20.00)	79.21 (22.68)	81.54 (20.45)	77.71 (20.05)	79.53 (13.61)	78.94 (18.22)	81.61 (20.65)	0.929
Mean (SD) SVC in %pred at baseline	76.52 (21.58)	76.24 (23.05)	83.29 (23.69)	75.11 (21.45)	70.70 (15.71)	73.01 (20.55)	80.53 (21.16)	0.232
Mean PCF (SD) at baseline	343.54 (128.86)	360.24 (128.98)	331.90 (159.99)	341.36 (143.36)	301.10 (88.74)	326.52 (124.16)	363.62 (115.50)	0.316
Mean SNIP (SD) at baseline	61.55 (27.91)	61.55 (27.91)	58.57 (24.32)	55.22 (26.99)	58.65 (27.93)	64.12 (31.77)	64.12 (31.77)	0.334
Median [IQR] ALSFRS-R respiratory at baseline	12.00 [11.00, 12.00]	12.00 [11.00, 12.00]	12.00 [11.00, 12.00]	12.00 [10.00, 12.00]	12.00 [11.00, 12.00]	12.00 [11.00, 12.00]	12.00 [11.00, 12.00]	0.325

*N*: number of participants; %: percentage of cohort; SD: standard deviation; IQR: interquartile range; ND: no data, date of onset not available.

* *p* Value of categorical variables calculated using Fisher’s exact test, Student’s *t*-test for normally distributed continuous variables, and the Kruskal–Wallis Rank Sum Test for non-normal continuous variables.

Raw forced vital capacity and SVC measurements were highly correlated (0.95), while peak cough flow had a moderate correlation with FVC (0.77) and SVC (0.77). SNIP was moderately correlated with FVC (0.55), SVC (0.56), and PCF (0.60) (Supplementary Table S1). The ALSFRS-R respiratory sub-score was poorly correlated with all respiratory measurements with correlations ranging from 0.30 to 0.36. The correlation between PCF and SNIP was higher in the bulbar sub-group (0.76). Correlations between the ALSFRS-R respiratory sub-score and respiratory measures were slightly higher in spinal patients (0.33–0.40) and lower in bulbar patients (0.17–0.22). These correlations were consistent with modeled correlations at six-monthly follow-up timepoints (Supplementary Table S2).

**Figure 1.**
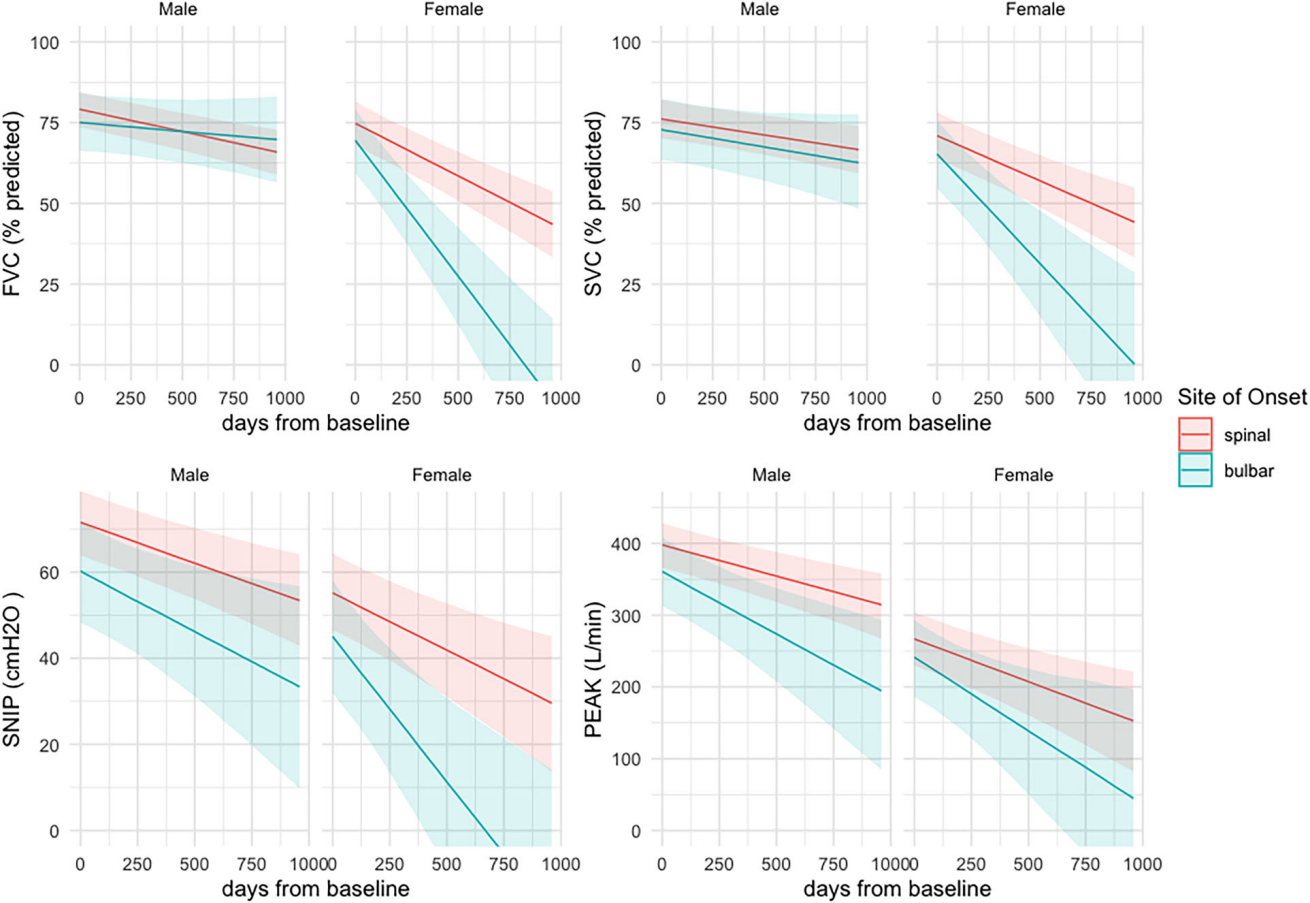
Conditional effect of site of onset and sex sampled from the multi-outcome Bayesian model using percent predicted FVC and SVC. Number of participants by sex and site of onset: male spinal-onset = 153, female spinal-onset = 64, male bulbar-onset = 27, female bulbar-onset = 24. FVC (%predicted): percent predicted of forced vital capacity as provided by GLI equations; SVC (%predicted): percent predicted of slow vital capacity based upon the GLI ratio for FVC; SNIP (cmH_2_O): sniff nasal inspiratory pressure in centimeters of water; PEAK (L/min): peak cough flow (liters per minute).

Figure 1 displays the decline in the respiratory measures stratified by sex and site of onset. In general, females have lower values in all measures and over time. The decline in females with bulbar onset disease is particularly steep, compared with males and with females with spinal onset disease. In males, FVC and SVC decline similarly in those with spinal- and bulbar-onset disease, while there is a large difference between the sub-groups in SNIP and PCF scores. Table 2 shows the modeled intercepts and slopes for the four respiratory outcome measures estimated by this model (Supplementary Table S3 and Figure S1 show the corresponding results using values in liters for FVC and SVC). Estimates for SNIP and PCF are minimally different, indicating that the transformation of FVC and SVC from liters to percent predicted has a minor impact on the overall model fit.

**Table 2.** Slopes and intercepts of respiratory measurements after Bayesian modeling of four outcomes using percent of predicted FVC and SVC.

		Intercept		Slope (unit decline per month)	
		Estimate	Credible interval (Q2.5, Q97.5)	Estimate	Credible interval (Q2.5, Q97.5)
*Males*					
FVC %pred	Spinal	79.3	(73.7, 85.0)	−0.42	(−0.58, −0.27)
	Bulbar	75.1	(61.2, 89.5)	−0.16	(−0.72, 0.39)
SVC % pred	Spinal	76.2	(70.3, 82.2)	−0.30	(−0.46, −0.14)
	Bulbar	72.8	(58.0, 87.8)	−0.32	(−0.92, 0.28)
SNIP (cmH_2_O)	Spinal	71.5	(63.9, 79.1)	−0.57	(−0.83, −0.31)
	Bulbar	60.1	(41.3, 79.2)	−0.84	(−1.84, 0.15)
PCF (L/min)	Spinal	398.2	(367.3, 429.7)	−2.65	(−3.83, −1.51)
	Bulbar	360.4	(284.5, 437.1)	−5.25	(−9.84, −0.78)
*Females*					
FVC %pred	Spinal	74.5	(63.1, 86.4)	−0.98	(−1.45, −0.51)
	Bulbar	69.4	(54.8, 84.3)	−2.57	(−3.56, −1.58)
SVC % pred	Spinal	70.8	(58.7, 83.1)	−0.85	(−1.35, −0.34)
	Bulbar	65.2	(49.7, 81.1)	−2.08	(−3.16, −1.01)
SNIP (cmH_2_O)	Spinal	55.2	(39.8, 70.7)	−0.80	(−1.58, −0.02)
	Bulbar	44.7	(25.1, 64.3)	−2.05	(−3.38, −0.70)
PCF (L/min)	Spinal	266.7	(202.4, 331.9)	−3.56	(−7.02, −0.06)
	Bulbar	239.8	(159.7, 321.4)	−6.27	(−12.42, −0.22)

FVC: forced vital capacity; SVC: slow vital capacity; %pred: percent predicted score provided by the GLI equations; SNIP: sniff nasal inspiratory pressure; cmH_2_O: centimeters of water (pressure); PCF: peak cough flow; L/min: liters per minutes; *Q*: quartile.

**Figure 2.**
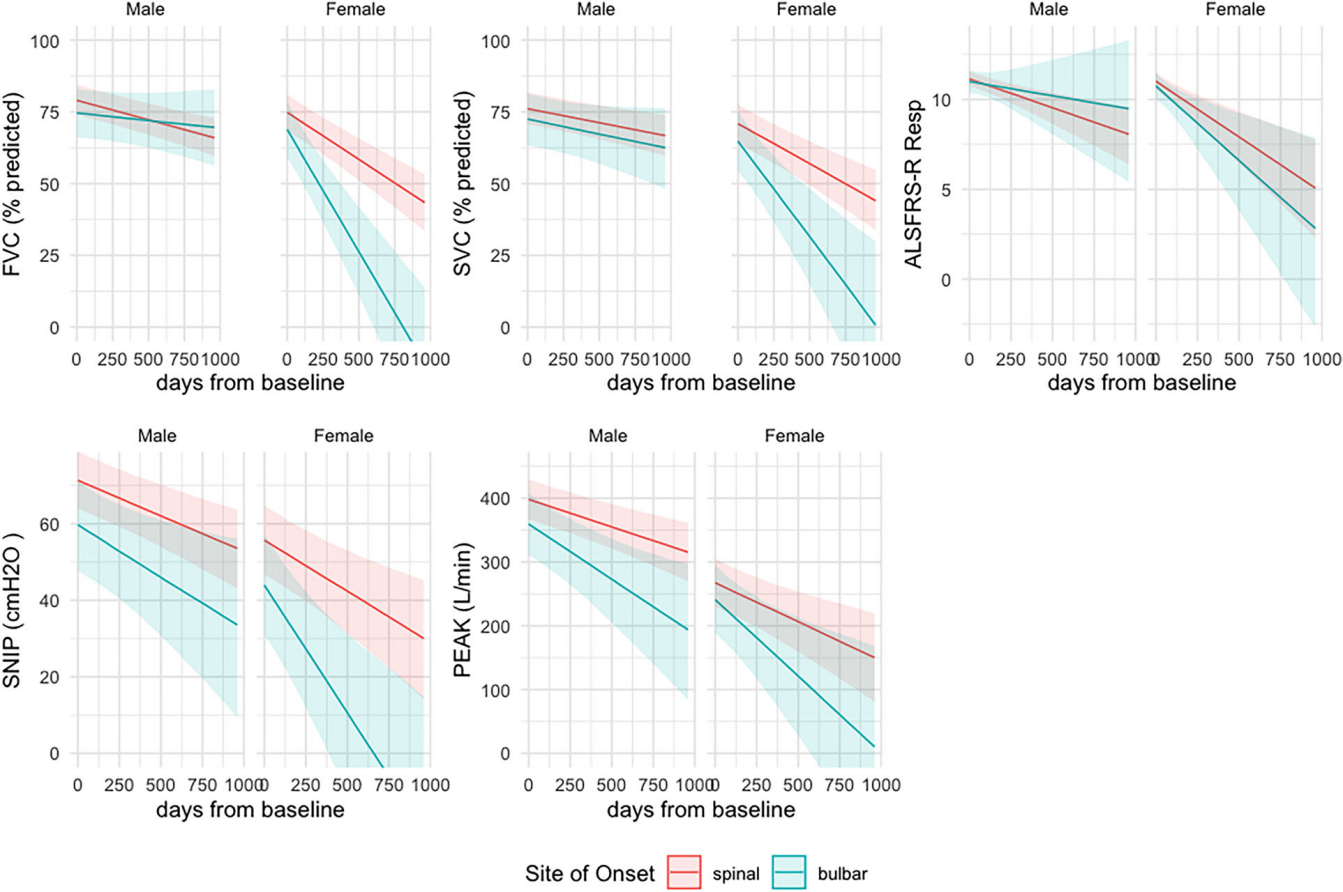
Conditional effect of site of onset and sex sampled from the multi-outcome Bayesian model using percent predicted FVC and SVC and extended to include the ALSFRS-R respiratory sub-score as an additional outcome. FVC (%predicted): percent predicted of forced vital capacity as provided by GLI equations; SVC (%predicted): percent predicted of slow vital capacity based upon the GLI ratio for FVC; SNIP (cmH_2_O): sniff nasal inspiratory pressure in centimeters of water; PEAK (L/min): peak cough flow (liters per minute).

Figure 2 shows the model with the ALSFRS-R respiratory sub-score included as a fifth outcome variable. The ALSFRS-R respiratory sub-score does not distinguish spinal and bulbar-onset disease in females as the four respiratory measurements do (the credible intervals for spinal vs. bulbar-onset fits overlap more completely than for the other outcomes). Table 3 shows the modeled intercepts and slopes for the five outcome measures estimated by the same model used to generate Figure 2. The equivalent for FVC and SVC in liters is shown in supplementary Table S4 and Figure S2. The estimates for SNIP and PCF have only minor differences between Table 3 and Table S4.

**Table 3.** Slopes and intercepts of respiratory measurements, including the ALSFRS-R respiratory sub-scale after Bayesian modeling of the 5 outcomes using percent predicted FVC and SVC.

		Intercept		Slope (unit decline per month)	
		Estimate	Credible interval (*Q*2.5, *Q*97.5)	Estimate	Credible interval (*Q*2.5, *Q*97.5)
*Males*					
FVC %pred	Spinal	78.9	(73.3, 84.3)	−0.41	(−0.56, −0.27)
	Bulbar	74.4	(60.0, 88.3)	−0.17	(−0.71, 0.38)
SVC % pred	Spinal	76.0	(69.9, 81.8)	−0.29	(−0.46, −0.14)
	Bulbar	72.2	(56.8, 87.0)	−0.32	(−0.92, 0.27)
SNIP (cmH_2_O)	Spinal	71.2	(63.6, 78.8)	−0.56	(−0.83, −0.29)
	Bulbar	59.4	(40.5, 78.3)	−0.83	(−1.88, 0.16)
PCF (L/min)	Spinal	396.8	(365.4, 428.0)	−2.60	(−3.82, −1.40)
	Bulbar	358.0	(280.4, 437.3)	−5.30	(−9.98, −0.70)
ALSFRS-R respiratory sub-score	Spinal	11.1	(10.7, 11.6)	−0.10	(−0.15, −0.05)
	Bulbar	11.0	(9.9, 12.0)	−0.04	(−0.24, 0.15)
*Females*					
FVC %pred	Spinal	74.4	(62.4, 86.1)	−0.99	(−1.45, −0.53)
	Bulbar	68.6	(53.9, 82.7)	−2.59	(−3.63, −1.58)
SVC % pred	Spinal	70.5	(57.8, 82.9)	−0.84	(−1.34, −0.35)
	Bulbar	64.4	(48.8, 79.5)	−2.01	(−3.14, −0.92)
SNIP (cmH_2_O)	Spinal	55.5	(39.6, 71.3)	−0.80	(−1.60, 0.00)
	Bulbar	43.8	(24.4, 63.0)	−2.04	(−3.48, −0.63)
PCF (L/min)	Spinal	266.8	(201.4, 333.2)	−3.61	(−7.15, −0.11)
	Bulbar	239.1	(157.7, 320.6)	−7.34	(−13.69, −0.93)
ALSFRS-R respiratory sub-score	Spinal	11.0	(10.2, 11.9)	−0.19	(−0.34, −0.04)
	Bulbar	10.7	(9.6, 11.8)	−0.25	(−0.49, −0.02)

FVC: forced vital capacity; SVC: slow vital capacity; %pred: percent predicted score provided by GLI equations; SNIP: sniff nasal inspiratory pressure; cmH_2_O: centimeters of water (pressure); PCF: peak cough flow; L/min: liters per minutes; *Q*: quartile.

Table 4 shows the results of models extended to include investigational explanatory variables. King’s stage 3 at baseline was associated with lower respiratory metrics across the course of follow-up when compared to King’s stage 2. However, a history of having ever smoked at baseline was not associated with differences in respiratory metrics, nor was having a history of respiratory tract infections at baseline associated with respiratory outcome measures. Orthopnea and dyspnea at rest and when active at baseline were all associated with reduced respiratory scores, with dyspnea at rest having the most negative parameter estimates. Having a regular productive cough at baseline was associated with a reduced PCF score.

**Table 4.** Regression coefficients for investigational explanatory variables.

	FVC % predicted			SVC % predicted		SNIP (cmH_2_O)					PCF (L/min)		ALSFRS-R		
Variable/strata (*N*)	Estimate	CrI: 2.5	CrI: 97.5	Estimate	CrI: 2.5	CrI: 97.5	Estimate	CrI: 2.5	CrI: 97.5	Estimate	CrI: 2.5	CrI: 97.5	Estimate	CrI: 2.5	CrI: 97.5
King’s stage 3 (vs. stage 2) at baseline	−9.1	−14.2	−4.12	−9.4	−14.75	−4.11	−8.8	−15.9	−1.9	−44.9	−73.3	−15.9	−0.7	−1.1	−0.3
Ever vs. never smoked at baseline	−1.7	−6.5	3.4	−2.3	−7.5	3.0	3.4	−3.4	10.2	−23.4	−52.8	4.98	0.1	−0.3	0.4
*Respiratory history at baseline*															
Occasional chest infections	0.9	−5.7	7.6	1.6	−5.4	8.7	3.5	−5.3	12.5	−11.0	−48.8	25.9	0.2	−0.3	0.7
Moderate/frequent chest infections	−1.5	−15.8	13.4	−2.9	−18.8	13.2	−5.0	−25.0	14.9	9.11	−72.2	94.3	−0.2	−1.3	0.9
*Respiratory symptoms at baseline*															
Orthopnea	−9.3	−17.1	−1.3	−10.3	−18.7	−1.8	−18.6	−29.3	−8.3	−43.9	−87.9	−0.3	−2.2	−2.7	−1.7
Dyspnea at rest	−18.5	−30.4	−6.5	−22.8	−35.8	−10.3	−18.6	−34.7	−2.2	−71.7	−140.5	−4.3	−2.6	−3.4	−1.8
Dyspnea when active	−13.3	−18.9	−7.3	−15.6	−21.7	−9.3	−15.3	−23.1	−7.2	−43.5	−77.0	−9.9	−1.5	−1.9	−1.1
Regular productive cough	−5.1	−10.8	1.0	−4.5	−10.7	2.00	−6.5	−14.4	1.3	−41.7	−74.5	−8.9	−0.5	−0.9	−0.0

## Discussion

This multicenter observational study analyzed the decline in commonly used respiratory measures (FVC, SVC, SNIP, PCF, and ALSFRS-R respiratory sub-score) in disease-onset and sex sub-groups and found significant differences between the groups. Females had lower scores in all measures at all timepoints and females with bulbar onset ALS had faster decline compared with all other sub-groups. Clinical symptoms recorded at baseline, including the presence of dyspnea and orthopnea and a higher King’s Stage were associated with lower scores on respiratory tests throughout follow-up, while having a regular productive cough at baseline was associated with a lower peak cough flow score.

The study recruited people in the middle stages of ALS (King’s stage 2 and 3), who were likely to experience respiratory decline, while being well enough to attend for assessment during follow-up. The percentage of participants with bulbar-onset disease was less than previously reported in similar European cohorts (30), which is possibly attributed to a relatively long delay from disease-onset to study entry.

Definition of differential trajectories of respiratory measurements when stratified by sex and site of onset in ALS patients is novel. Females had lower scores in all measures at all timepoints, which is particularly apparent using raw SVC and FVC scores, but is also evident using percent predicted scores (accommodating sex, height, and age). Bulbar-onset disease is widely recognized as more common in females and associated with a shorter prognosis (31). Different patterns of decline in males and females with bulbar-onset disease are clearly shown with a steep decline in females with bulbar weakness compared with other sub-groups (Figure 1). Peak cough flow and SNIP measurements show steeper decline in males with bulbar-onset ALS compared with males with spinal-onset, while the pattern of decline in FVC and SVC is similar. The markedly different pattern of decline in males and females with bulbar-onset ALS, particularly in SVC and FVC is likely to reflect differences in the manifestations of the disease on the respiratory system in males and females. Interestingly, the pattern of decline in peak cough flow is similar in males and females overall, while SNIP also declines more steeply in females with bulbar-onset ALS.

A number of clinical prognostic indicators were examined, demonstrating that King’s stage 3 at baseline (i.e. the disease was more progressed) was associated with lower respiratory scores throughout follow-up compared with King’s stage 2. In addition, we have shown that clinical symptoms at baseline including orthopnea and dyspnea at rest and when active were associated with lower respiratory scores, while having a history of regular productive cough was only associated with a lower peak cough flow score. Causality cannot be determined, but it is likely that weakness of the respiratory musculature and of cough strength results in secretion retention and increased awareness of the patient of the need to clear secretions. This validates the use of screening questions on cough, orthopnea, and dyspnea in clinical practice as indicators of respiratory weakness. However, this association does not translate to a correlation between the objective respiratory measurements and the respiratory sub-scale of the ALSFRS-R, which was weak (*r* = 0.3–0.36), which may be due to the use of arbitrary ranking in the scale. A smoking history at baseline was not associated with respiratory weakness, likely due to the effect of ALS on respiratory function being much larger than any respiratory effect of smoking. Other potential predictive features such as ethnicity and BMI were not addressed in this study but warrant investigation in future studies.

The ALSFRS-R respiratory sub-score is poorly correlated with all four objective respiratory measurements particularly in bulbar-onset patients, which is similar to previous reports (32). The ongoing use of the ALSFRS-R as a global outcome in clinical trials has been regarded as problematic by many authors (8,33,34) and the poor associations further emphasize this issue.

In our interim analysis paper (19), we assessed the correlations between the respiratory measures and reported an almost perfect correlation between FVC and SVC at baseline, which persisted over time, in agreement with previous reports (35). The primary variable of interest in ALS provided by FVC or SVC is the volume of air exhaled, which is likely to be similar regardless of the technique used. SVC is suggested as more appropriate for patients with bulbar-onset ALS, as it does not require a rapid and forceful exhalation, which can be challenging. We collected both SVC and FVC in this study using a facemask from baseline. This was to ensure that issues that were likely to have arisen during the study resulting from an inability to achieve a mouth seal due to evolving bulbar weakness were avoided. This likely contributed to the close correlation between SVC and FVC, but also to the ability of participants to complete the vital capacity tests during follow-up.

The range of estimates for decline in respiratory scores reported in our study is similar to previous reports (Table 5). For PCF, the slope obtained by Rafiq et al. (39) of −5.77 L/min/month falls between our slowest and fastest sub-groups, while that of Tattersall et al. (36) showed a steeper decline at −10.4 L/min/month, which may be explained by the inclusion of participants with more advanced disease. The slope of FVC percent predicted has been reported by Pinto and de Carvalho (35) who found a slope of −2.1%/month. SVC percent predicted on the other hand was reported by three studies (35–37), two of which (35,37) showed faster decline than our fastest sub-group. The slope of SNIP was only reported in two studies (36,38), one of which showed a faster decline than our fastest sub-group (38). Percentage change over two timepoints a mean of 5.2 months apart was reported by another study, which showed a 13.52% decline in SNIP, a 14% decline in ALSFRS-r, and 10.49% decline in FVC (40). The slope of the ALSFRS-R respiratory sub-score was only reported by one of these studies (35) and again was within the range spanned by our slowest and fastest groups.

**Table 5.** Slopes of respiratory metrics from comparable studies.

	Tattersall et al. (36) *n* = 106	Andrews et al. (37) *n* = 893	Pinto and de Carvalho (35) *n* = 592	Enache et al. (38) *n* = 256	Rafiq et al. (39) *n* = 21	Present study^a^ (slowest sub-group)	Present study^a^ (fastest sub-group)
PCF (L/min)	−10.4	–	–		−5.77	−2.62	−7.32
FVC %pred			−2.1			−0.16	−2.59
SVC %pred	−1.5	−2.7	−2.2			−0.30	−2.03
SNIPcmH_2_O	−1.6			−3.0		−0.57	−2.05
ALSFRS-R total	−0.81		0.9				
ALSFRS-R respiratory sub-scale			−0.15			−0.05	−0.25

^a^ Data taken from Table 3 indicate that the subgroup comprised of females with bulbar onset disease was the fastest progressing subgroup for all measures. Males with spinal onset disease were the subgroup whose SVC, PCF, and SNIP progressed most slowly, while males with bulbar onset disease had the lowest slope for FVC and ALSFRS-R respiratory subscale.

Limitations of the study include the curtailment of follow-up during the Covid-19 pandemic. Only two sites Leuven and Utrecht were able to collect respiratory measures in-person after February 2020. It is not possible to evaluate the impact of Covid-19 on the respiratory function of participants as Covid-19 infection was not specifically collected. In addition, we were unable to extend the models to allow for non-linear decline, as this requires a larger dataset. Future studies utilizing larger datasets, which include detailed respiratory and clinical data collected should replicate the analysis and consider non-linear patterns of decline (41). The findings are applicable to the middle stages of ALS (Kings stage 2 and 3) and future studies should examine the findings across all stages of ALS. Future work using the current dataset will include analysis of the frequency and impact of respiratory tract infections and analysis of the association between reported symptoms and morbidity and respiratory test scores.

## Conclusion

Using Bayesian methods, we have shown that respiratory function declines more quickly in female ALS patients when compared to males when measured by FVC, SVC, SNIP, or PCF, but not the ALSFRS-R respiratory sub-score. Furthermore, females with bulbar onset ALS decline fastest. In addition, we showed that the presence of baseline clinical symptoms including breathlessness in lying, dyspnea at rest and dyspnea when active and a higher King’s staging at baseline were associated with lower respiratory scores over time. Furthermore, we showed that the ALSFRS-R respiratory sub-score is poorly correlated with objective respiratory measurements.

## Supplementary Material

Supplemental Material
