# Supplementary material for: REVEALS—a longitudinal cohort study of multifaceted respiratory assessment in ALS
Source: Amyotroph Lateral Scler Frontotemporal Degener. 2024 Jun 6;25(7-8):661–71. doi: 10.1080/21678421.2024.2359556 (PMC11523912; doi:10.1080/21678421.2024.2359556)
Supplement: Supplemental Material [file IAFD_A_2359556_SM9001.docx]

**Supplementary Material**

**Supplementary Table S1. Crude correlations of respiratory measurement scores in all patients and stratified by site of onset**

| **All patients** | | | | | |  |
| --- | --- | --- | --- | --- | --- | --- |
|  | **FVC** | **SVC** | **PCF** | **SNIP** | **ALSFRS-R Resp** | |
| **FVC** | 1.0 | 0.95 | 0.77 | 0.55 | | 0.31 |
| **SVC** | 0.95 | 1.0 | 0.77 | 0.56 | | 0.32 |
| **PCF** | 0.77 | 0.77 | 1.0 | 0.60 | | 0.30 |
| **SNIP** | 0.55 | 0.56 | 0.60 | 1.0 | | 0.36 |
| **ALSFRS-R Resp** | 0.31 | 0.32 | 0.30 | 0.36 | | 1.0 |
| **Spinal-onset only** | | | | | |  |
|  | **FVC** | **SVC** | **PCF** | **SNIP** | **ALSFRS-R Resp** | |
| **FVC** | 1.0 | 0.95 | 0.78 | 0.53 | | 0.33 |
| **SVC** | ﻿0.95 | 1.0 | 0.78 | 0.54 | | 0.35 |
| **PCF** | ﻿0.78 | ﻿0.78 | 1.0 | 0.55 | | 0.33 |
| **SNIP** | ﻿0.53 | ﻿0.54 | ﻿0.55 | 1.0 | | 0.40 |
| **ALSFRS-R Resp** | 0.33 | 0.35 | 0.33 | 0.40 | | 1.0 |
| **Bulbar-onset only** | | | | | |  |
|  | **FVC** | **SVC** | **PCF** | **SNIP** | **ALSFRS-R Resp** | |
| **FVC** | 1.0 | 0.95 | 0.71 | 0.60 | | 0.22 |
| **SVC** | ﻿0.95 | 1.0 | 0.72 | 0.60 | | 0.21 |
| **PCF** | ﻿0.71 | ﻿0.72 | 1.0 | 0.76 | | 0.18 |
| **SNIP** | ﻿0.60 | ﻿0.60 | 0.76 | 1.0 | | 0.17 |
| **ALSFRS-R Resp** | 0.22 | 0.21 | 0.18 | 0.17 | | 1.0 |

FVC: Forced Vital Capacity, SVC: Slow Vital Capacity, PCF: Peak cough flow SNIP: Sniff Nasal Inspiratory Pressure, ALSFRS-R Resp: respiratory sub-score of the ALSFRS-R (i.e. the sum of questions 10, 11 & 12)

**Supplementary Table S2. Bayesian model predicted correlations at six-monthly follow-up times.**

| Baseline |  |  |  |  |  |
| --- | --- | --- | --- | --- | --- |
|  | **FVC** | **SVC** | **SNIP** | **PCF** | **ALSFRS-R Resp** |
| FVC | 1.00 | 0.95 | 0.57 | 0.76 | 0.31 |
| SVC | 0.95 | 1.00 | 0.58 | 0.77 | 0.31 |
| SNIP | 0.57 | 0.58 | 1.00 | 0.61 | 0.35 |
| PCF | 0.76 | 0.77 | 0.61 | 1.00 | 0.28 |
| ALSFRS-R Resp | 0.31 | 0.31 | 0.35 | 0.28 | 1.00 |
| 6 months |  |  |  |  |  |
|  | **FVC** | **SVC** | **SNIP** | **PCF** | **ALSFRS-R Resp** |
| FVC | 1.00 | 0.95 | 0.60 | 0.77 | 0.36 |
| SVC | 0.95 | 1.00 | 0.60 | 0.78 | 0.36 |
| SNIP | 0.60 | 0.60 | 1.00 | 0.63 | 0.38 |
| PCF | 0.77 | 0.78 | 0.63 | 1.00 | 0.33 |
| ALSFRS-R Resp | 0.36 | 0.36 | 0.38 | 0.33 | 1.00 |
| 12 months |  |  |  |  |  |
|  | **FVC** | **SVC** | **SNIP** | **PCF** | **ALSFRS-R Resp** |
| FVC | 1.00 | 0.95 | 0.61 | 0.76 | 0.34 |
| SVC | 0.95 | 1.00 | 0.62 | 0.77 | 0.34 |
| SNIP | 0.61 | 0.62 | 1.00 | 0.63 | 0.35 |
| PCF | 0.76 | 0.77 | 0.63 | 1.00 | 0.30 |
| ALSFRS-R Resp | 0.34 | 0.34 | 0.35 | 0.30 | 1.00 |
| 18 months |  |  |  |  |  |
|  | **FVC** | **SVC** | **SNIP** | **PCF** | **ALSFRS-R Resp** |
| FVC | 1.00 | 0.95 | 0.61 | 0.73 | 0.31 |
| SVC | 0.95 | 1.00 | 0.61 | 0.74 | 0.31 |
| SNIP | 0.61 | 0.61 | 1.00 | 0.59 | 0.29 |
| PCF | 0.73 | 0.74 | 0.59 | 1.00 | 0.25 |
| ALSFRS-R Resp | 0.31 | 0.31 | 0.29 | 0.25 | 1.00 |

FVC: Forced Vital Capacity, SVC: Slow Vital Capacity, PCF: Peak cough flow SNIP: Sniff Nasal Inspiratory Pressure, ALSFRS-R Resp: respiratory sub-score of the ALSFRS-R (i.e. the sum of questions 10, 11 & 12)

**Supplementary Table S3. Slopes and intercepts of respiratory measurements after Bayesian modelling of 4 outcomes using absolute values of FVC and SVC**

|  |  | **Intercept** | | **Slope**  **(unit decline per month)** | |
| --- | --- | --- | --- | --- | --- |
|  |  | **Estimate** | **Credible Interval**  **(Q2.5, Q97.5)** | **Estimate** | **Credible Interval**  **(Q2.5, Q97.5)** |
| **Males** | | | | | |
| **FVC (L)** | **Spinal** | 3.44 | (3.19, 3.70) | -0.02 | (-0.03, -0.02) |
|  | **Bulbar** | 3.29 | (2.68, 3.91) | -0.01 | (-0.03, 0.01) |
| **SVC (L)** | **Spinal** | 3.31 | (3.06, 3.57) | -0.02 | (-0.02, -0.01) |
|  | **Bulbar** | 3.20 | (2.58, 3.84) | -0.02 | (-0.04, 0.01) |
| **SNIP (cmH_2_O)** | **Spinal** | 72.27 | (64.65, 79.88) | -0.54 | (-0.81, -0.28) |
|  | **Bulbar** | 60.14 | (41.06, 79.19) | -0.71 | (-1.71, 0.27) |
| **PCF (L/min)** | **Spinal** | 402.22 | (371.68, 436.02) | -2.40 | (-3.54, -1.31) |
|  | **Bulbar** | 358.74 | (281.15, 436.02) | -3.93 | (-8.07, 0.15) |
| **Females** | | | | | |
| **FVC (L)** | **Spinal** | 2.25 | (1.73, 2.78) | -0.03 | (-0.05, -0.01) |
|  | **Bulbar** | 2.02 | (1.35, 2.68) | -0.08 | (-0.12, -0.04) |
| **SVC (L)** | **Spinal** | 2.13 | (1.61, 2.66) | -0.03 | (-0.05, -0.01) |
|  | **Bulbar** | 1.88 | (1.21, 2.56) | -0.07 | (-0.11, -0.02) |
| **SNIP (cmH2O)** | **Spinal** | 56.40 | (40.80, 72.12) | -0.77 | (-1.56, 0.03) |
|  | **Bulbar** | 44.97 | (25.92, 64.19) | -2.06 | (-3.40, -0.70) |
| **PCF (L/min)** | **Spinal** | 269.72 | (206.54, 335.66) | -3.45 | (-6.69, -0.19) |
|  | **Bulbar** | 241.50 | (160.54, 323.88) | -6.38 | (-12.27, -0.45) |

FVC(L) = Absolute score in Litres for Forced Vital Capacity. SVC(L) Absolute score in Litres for Slow Vital Capacity. SNIP(cmH2O) = Sniff Nasal Inspiratory Pressure in centimetres of water. PCF (L/min) = Peak Cough Flow in Litres per minute. Q= quartile.

**Supplementary Figure S1.**


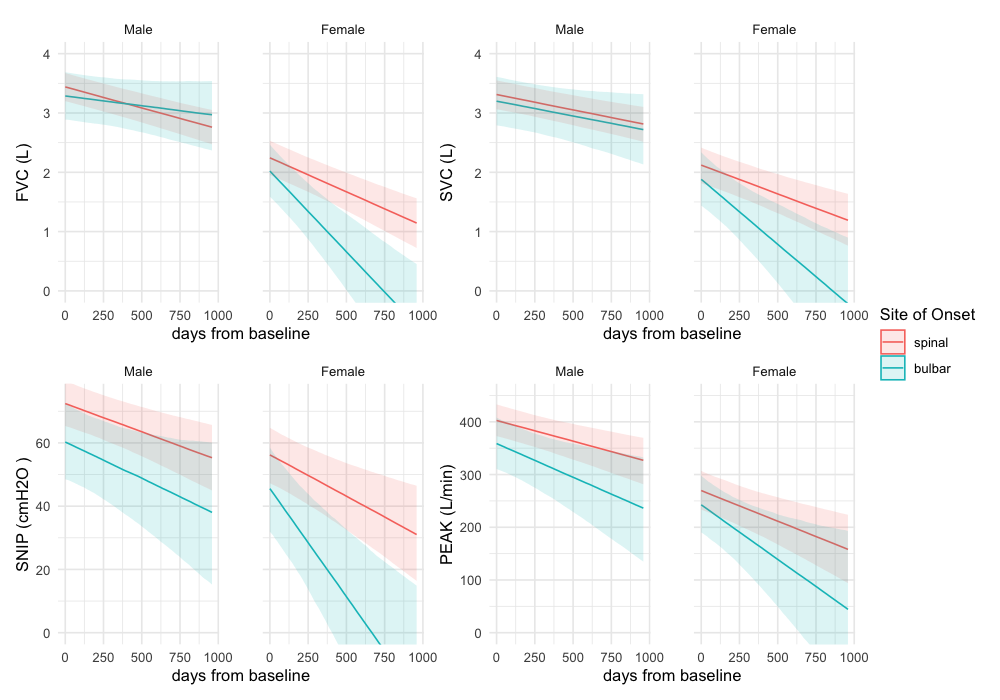


Supplementary Figure S1.

Legend:

Number of participants by sex and site of onset: male spinal-onset = 153, female spinal-onset = 64, male bulbar-onset = 27, female bulbar-onset = 24. FVC(L) = Absolute score in Litres for Forced Vital Capacity. SVC(L)) Absolute score in Litres for Slow Vital Capacity. SNIP(cmH_2_O) = Sniff Nasal Inspiratory Pressure in centimetres of water. PEAK(L/min) = Peak Cough Flow in Litres per minute.

**Supplementary Table 4. Slopes and intercepts of respiratory measurements after Bayesian multiple outcome modelling of 5 outcomes and using absolute values in Litres of FVC and SVC**

|  |  | Intercept | | Slope /month or day | |
| --- | --- | --- | --- | --- | --- |
|  |  | Estimate | Credible Interval  (Q2.5, Q97.5) | Estimate | Credible Interval  (Q2.5, Q97.5) |
| Males | | | | | |
| FVC (L) | Spinal | 3.42 | (3.17, 3.66) | -0.02 | (-0.03, -0.01) |
|  | Bulbar | 3.29 | (2.66, 3.90) | -0.01 | (-0.03, 0.01) |
| SVC (L) | Spinal | 3.29 | (3.04, 3.54) | -0.02 | (-0.02, -0.01) |
|  | Bulbar | 3.21 | (2.57, 3.83) | -0.02 | (-0.04, 0.01) |
| SNIP (cmH_2_O) | Spinal | 71.9 | (64.5, 79.55) | -0.54 | (-0.80, -0.28) |
|  | Bulbar | 60.2 | (41.4, 78.8) | -0.69 | (-1.68, 0.28) |
| PCF (L/min) | Spinal | 400.1 | (369.7, 431.0) | -2.37 | (-3.52, -1.27) |
|  | Bulbar | 359.3 | (282.2, 437.0) | -4.00 | (-8.22, 0.14) |
| ALSFRS-R respiratory sub-score | Spinal | 11.2 | (10.8, 11.6) | -0.10 | (-0.15, -0.04) |
|  | Bulbar | 11.0 | (10.0, 12.0) | -0.04 | (-0.23, 0.16) |
| Females | | | | | |
| FVC (L) | Spinal | 2.26 | (1.74, 2.78) | -0.03 | (-0.05, -0.01) |
|  | Bulbar | 1.99 | (1.34, 2.63) | -0.09 | (-0.13, -0.04) |
| SVC (L) | Spinal | 2.13 | (1.61, 2.65) | -0.03 | (-0.05, -0.01) |
|  | Bulbar | 1.86 | (1.19, 2.52) | -0.07 | (-0.11, -0.02) |
| SNIP (cmH2O) | Spinal | 56.7 | (41.2, 72.3) | -0.77 | (-1.54, 0.01) |
|  | Bulbar | 44.4 | (24.9, 64.2) | -2.06 | (-3.49, -0.67) |
| PCF (L/min) | Spinal | 270.4 | (206.7, 334.7) | -3.50 | (-6.82, -0.22) |
|  | Bulbar | 240.1 | (160.7, 320.7) | -7.43 | (-13.71, -1.14) |
| ALSFRS-R respiratory sub-score | Spinal | 11.0 | (10.2, 11.9) | -0.19 | (-0.34, -0.03) |
|  | Bulbar | 10.8 | (9.7, 11.9) | -0.25 | (-0.49, -0.01) |

FVC(L) = Absolute score in Litres for Forced Vital Capacity. SVC(L) Absolute score in Litres for Slow Vital Capacity. SNIP(cmH2O) = Sniff Nasal Inspiratory Pressure in centimetres of water. PCF (L/min) = Peak Cough Flow in Litres per minute.


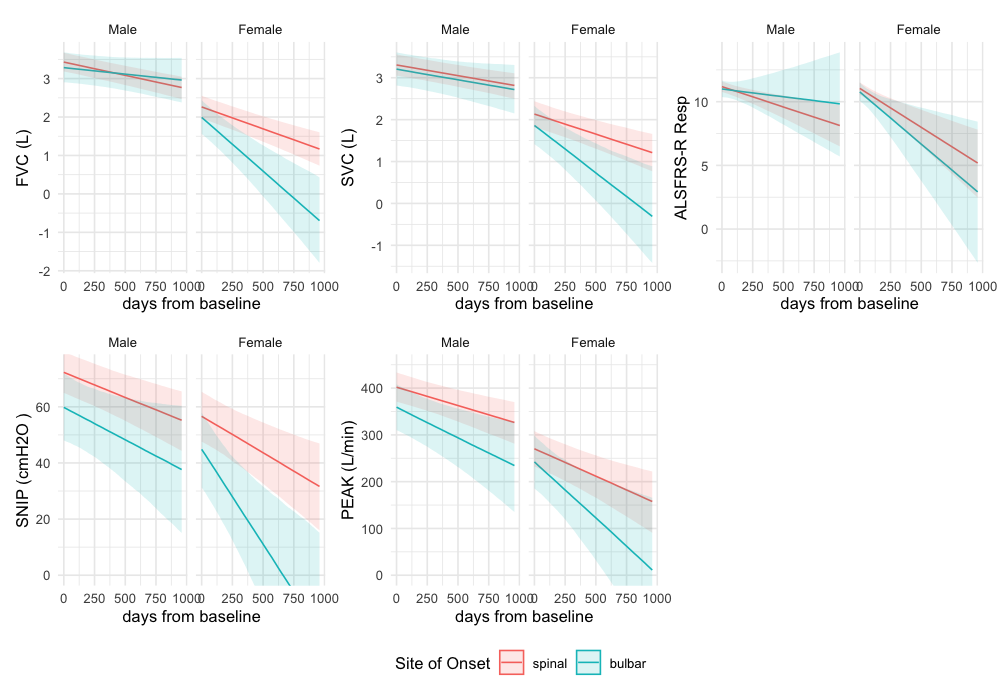


Supplementary Figure S2.

Legend:

FVC(L) = Absolute score in Litres for Forced Vital Capacity. SVC(L) Absolute score in Litres for Slow Vital Capacity. SNIP (cmH2O) = Sniff Nasal Inspiratory Pressure in centimetres of water. PEAK(L/min) = Peak Cough Flow in Litres per minute.

Supplementary Material Figure Caption (List)

Supplementary Figure S1. Conditional effect of site of onset and sex sampled from the 4 outcome Bayesian model using absolute values of FVC and SVC

Supplementary Figure S2. Conditional effect of site of onset and sex sampled from the 5 outcome Bayesian model using absolute values of FVC and SVC measured in Litres
